# Disruption of male fertility-critical *Dcaf17 dysregulates mouse testis transcriptome*

**DOI:** 10.1038/s41598-022-25826-7

**Published:** 2022-12-12

**Authors:** Raed Abu-Dawud, Bhavesh V. Mistry, Mohamed Rajab, Maha Alanazi, Nadya Al-Yacoub, Junaid Kashir, Falah Almohanna, Dilek Colak, Abdullah M. Assiri

**Affiliations:** 1 Department of Comparative Medicine, King Faisal Specialist Hospital & Research Centre, P.O. Box 3354, Riyadh, 11211 Saudi Arabia; 2 Department of Molecular Oncology, King Faisal Specialist Hospital & Research Centre, Riyadh, 11211 Saudi Arabia; 3 College of Medicine, Alfaisal University, Riyadh, 11533 Saudi Arabia

**Keywords:** Transcriptomics, Proteolysis, Ubiquitin ligases, Ubiquitylation, Spermatogenesis

## Abstract

During mammalian spermatogenesis, the ubiquitin proteasome system maintains protein homoeostasis (proteastasis) and spermatogenic cellular functions. DCAF17 is a substrate receptor in the ubiquitin CRL4 E3 Ligase complex, absence of which causes oligoasthenoteratozoospermia in mice resulting in male infertility. To determine the molecular phenomenon underlying the infertility phenotype caused by disrupting *Dcaf17*, we performed RNA-sequencing-based gene expression profiling of 3-weeks and 8-weeks old *Dcaf17* wild type and *Dcaf17* disrupted mutant mice testes. At three weeks, 44% and 56% differentially expressed genes (DEGs) were up- and down-regulated, respectively, with 32% and 68% DEGs were up- and down-regulated, respectively at 8 weeks. DEGs include protein coding genes and lncRNAs distributed across all autosomes and the X chromosome. Gene ontology analysis revealed major biological processes including proteolysis, regulation of transcription and chromatin remodelling are affected due to *Dcaf17* disruption. We found that *Dcaf17* disruption up-regulated several somatic genes, while germline-associated genes were down-regulated. Up to 10% of upregulated, and 12% of downregulated, genes were implicated in male reproductive phenotypes. Moreover, a large proportion of the up-regulated genes were highly expressed in spermatogonia and spermatocytes, while the majority of downregulated genes were predominantly expressed in round spermatids. Collectively, these data show that the *Dcaf17* disruption affects directly or indirectly testicular proteastasis and transcriptional signature in mouse.

## Introduction

Mammalian testicular function requires coordinated multifaceted transcriptional and translational control mechanisms facilitated by intimate germ and somatic cell communication under the control of the hypothalamic-pituitary–gonadal axis. Spermatogenesis involves many molecular processes, which are accompanied by controlled proteastasis to permit normal sperm development. Disruption of proteostasis, i.e., by disruption of the ubiquitination pathways, can cause spermatogenic failure leading to infertility^1–4^.

Ubiquitination is a post-translational modification which determines the target protein‘s fate and/or function via proteolytic and non-proteolytic processes. Mono- or poly-ubiquitination of protein substrates occur via successive actions of E1 ubiquitin-activating enzyme, E2 ubiquitin-conjugating enzyme and E3 ubiquitin ligase enzyme^5,6^. The E3 ligases are a large group of proteins with important roles in biological processes including spermatogenesis^7,8^. The cullin-RING E3 ligase (CRL4) is considered the largest family of the E3 ligases, which is abundantly expressed in testis^7^. It is thought that CRL4 ligases exert precise tissue specific functions through partnering with Ddb1 and Cul4-Associated Factors (DCAFs)^9^. There are over 60 known *DCAFs* in human and mouse that serve as substrate receptors in the CRL4 complex and shown to be highly expressed in the testis, perhaps playing an important role in spermatogenesis^10^.

*Dcaf17* is a putative substrate receptor, which facilitates the ubiquitination of target proteins by the DDB1-Cul4 E3 ligase complex. *Dcaf17*, a component of an E3 ligase ubiquitination complex, is essential for normal spermatogenesis, as its disruption in mouse causes male infertility due to abnormal sperm morphology, abolished motility, and low count, resulting in oligoasthenoteratozoospermia^11^.

To understand the molecular mechanisms underlying the spermatogenic defect in the *Dcaf17* knock-out (KO) model, RNAseq based gene expression profiling was performed using *Dcaf17* mutant and wild type testes of sexually immature (3 weeks) and mature (8 weeks) mice. Our data show the up-regulation of genes of somatic tissues and the down-regulation of germline specific genes in the mutant. This perturbed gene expression profile might be due to aberrant proteolytic and/or non-proteolytic ubiquitination pathway, which also govern chromatin state alterations and dysregulation of transcription. This study sheds light on the function of E3 ligase substrate receptor, *Dcaf17*, and warrants further investigation on its role in the crosstalk between ubiquitination and gene expression regulation in the testis.

## Results

### Testicular RNA-seq-based gene expression profiling in *Dcaf17* wild type and KO Mice

We have previously shown that the *Dcaf17* KO male mice are infertile and display reduced sperm counts, reduced sperm motility and dysmorphic sperm^11^. To gain detailed insights into the underlying mechanisms of this phenotype, we carried out RNA-Seq-based gene expression signatures of testis of mutants and wild types taken from 3 weeks (21 days) and 8 weeks (56 days) old mice. There are three reasons for this age choice. First, the expression for *Dcaf17* starts at day 20^11^. Second, we previously showed that Dcaf17 disruption mainly affects spermiogenesis, 3 weeks and 8 weeks of age allows to assess before and after spermiogenesis^10^. Third, it was reported that Dcaf17 is uniquely present at the onset of round spermatid development^12^. Three biological replicates each were run using the Ion AmpliSeq Mouse Transcriptome panel that represents more than 20,000 well-annotated RefSeq genes and predicted RNA transcripts. The run produced over 77 million reads with high (> 99%) mapping rate to RNA targets. However, three samples did not pass the quality control resulting in further analysis of two biological replicates of 3 weeks WT, 3 weeks mutant and 8 weeks mutant, and three biological replicates of the 8 weeks WT.

**Figure 1:**
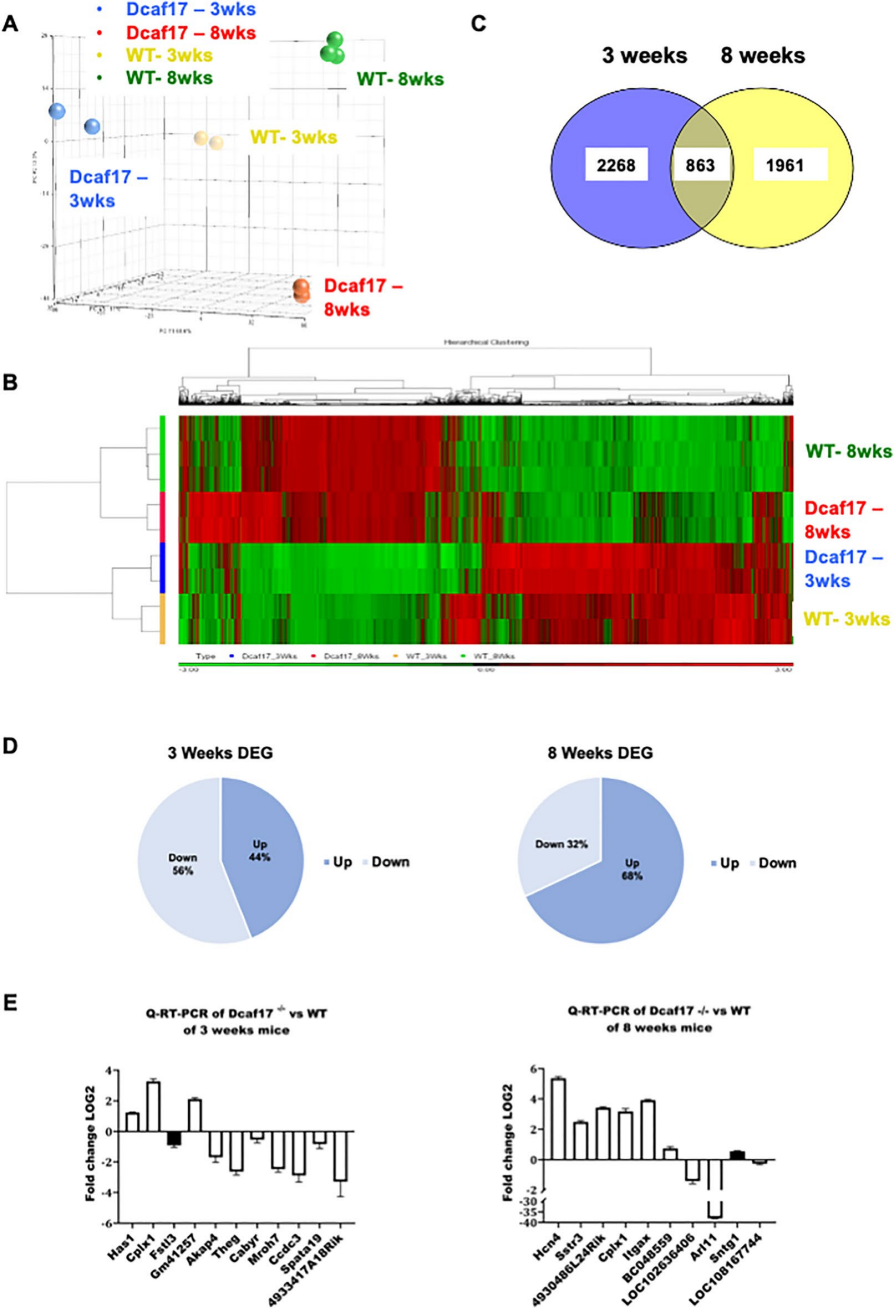
Quality control of AmpliSeq experiment for both age groups, 3 and 8 weeks, as well as for WT and mutants. (**A**) PCA plot and (**B**) heat map illustrate the cluster relationships. Genes clustered according to their age group and *Dcaf17* mutation status applying a differential expression *p-*value of > 0.05. Noteworthy, in spite of the severe male infertility phenotype the cluster separation in the PCA plot and the differences in the heatmap between the WT and mutants are not as dramatic as anticipated, strongly indicating that the small expression differences are critical for fertility, and potentially suggesting that Dcaf17 impacts the proteome more than the transcriptome. (**C**) Venn diagram showing the numbers of overlapping and distinct expressed genes. (**D**) Proportions of up- and down-regulated genes in *Dcaf17* mutant mice compared to WT in testicular tissues. (**E**) Q-PCR verification of the DEGs identified by the AmpliSeq of 3 (left) and 8 (right) weeks old *Dcaf17* mutant mice in comparison to the WT. In white, genes that follow the trend of the AmpliSeq data and, in black, genes that follow the opposite trend (Fstl3 and Sntg1). Note, all genes were verified to be differentially regulated. *Has1*, *Cplx1*, *Gm41257* were up-regulated in 3 weeks of age, and *Fstl3*, *Akap4*, *Theg*, *Cabyr*, *Mroh7*, *Ccdc3*, *Spata19* and *4933417A18Rik* were down-regulated. At 8 weeks of age *Hcn4*, *Sstr3*, *4930486L2Rik*, *Cplx1*, *Itgax*, *BC048559* and *Sntg1* were up-regulated, and *LOC102636406*, *Arl11* and *Loc108167744* were down-regulated. The statistical analysis for the Q-PCR are represented as the mean ± standard error of the mean (SEM). All the experiments were performed in triplicates of three biological replicates each. All statistical tests were two-sided and *p-*value < 0.05 was considered statistically significant.

The unsupervised principal component analysis (PCA) and hierarchical clustering revealed genes that display variation across samples (~ 15,000) and separated the samples according to their age group. Additionally, the PCA separated samples according to *Dcaf17* mutation status supporting the assertion of distinct gene expression changes associated with the mutation as well as with age (Fig. 1A). Noteworthy, is that despite the severe male infertility phenotype of the *Dcaf17* mutants the separation in the PCA plot and in the heat map (Fig. 1A, B) is not as dramatic as anticipated, therefore, highlighting the critical importance of the DEGs for male fertility, and potentially suggesting that Dcaf17 impacts the proteome more severely than the transcriptome.

### Transcriptomic signatures of *Dcaf17* wild type versus KO mice

The total number of differentially expressed genes (DEGs) in the 3-weeks age group is 3131 compared to 2824 in the 8-weeks-old mice, with 863 overlapping DEGs. 2268 DEGs were specific to the 3-week and 1961 DEGs specific to the 8 week age group (Fig. 1C and Supplementary Table 1). Approximately 68% of genes were up-regulated in the 8 weeks’ group (1921 up-regulated vs. 903 down-regulated genes), whereas, in the 3 weeks’ group, 56% of DEGs were down-regulated (1390 up- regulated vs. 1741 down-regulated genes) (Fig. 1D, Supplementary Tables 2 and 3). The up-regulated genes in both age groups contain genes related to somatic tissue functions, while down-regulated genes were enriched with male gonad transcriptome signatures (Supplementary Tables 2 and 3). Interestingly, among the overlapping genes, 526 (~ 61%) genes are following the same expression trend, while 337 (~ 39%) genes follow opposite trends. DAVID analysis of the genes that underwent a fold-change that is greater than 1.5, revealed gene enrichment for the biological processes of spermatogenesis, positive regulation of acrosome reaction, fusion of sperm to egg plasma membrane, estrous cycle, ion transport, fertilization and lipid metabolic process. In regards to the GO-Term Cellular Component, we observed an enrichment for acrosomal vesicle, acrosomal membrane and membrane (Supplemental Table 1).

### Validation of AmpliSeq data using Q-PCR

Q-PCR was carried out on a select group of genes from the Top 10 and Top 11 DEGs of each examined developmental stage, 3 and 8 weeks, to verify the RNA sequencing results obtained from the Ion AmpliSeq platform and to serve as a second quality control step. We confirmed the up-regulation of *Has1*, *Cplx1*, *Gm41257* and the down-regulation of *Akap4*, *Cabyr*, *Theg*, *Ccdc33*, *Mroh7*, *4933417A18Rik* and *Spata19* in 3 weeks *Dcaf17* disrupted mice (Fig. 1E). In the 8 weeks age group, the up-regulation of *Hcn4*, *4930486L24Rik*, *Cplx1*, *Sstr3*, *Itgax*, *BC048559* was detected in mutant mice compared to the wild type and down-regulation of *Arl11*, *LOC108167744* and *LOC102636406* was confirmed, which is in agreement with Ion AmpliSeq data (Fig. 1E).

Collectively, we have verified 90% (18 out of 20) of genes that are in concordance with the Ion AmpliSeq data using Q-PCR, except for *Sntg1* and *Fstl3* that showed opposite trends (Fig. 1E). This discrepancy between the Ion AmpliSeq data and the Q-PCR is perhaps due to the different methods used, which remains, however, to be clarified experimentally. Nonetheless, in agreement with the Ion AmpliSeq data, all 20 examined genes were differentially expressed at 3 and 8 weeks of age.

### Dysregulation of gene expression caused by the *Dcaf17* disruption

**Figure 2:**
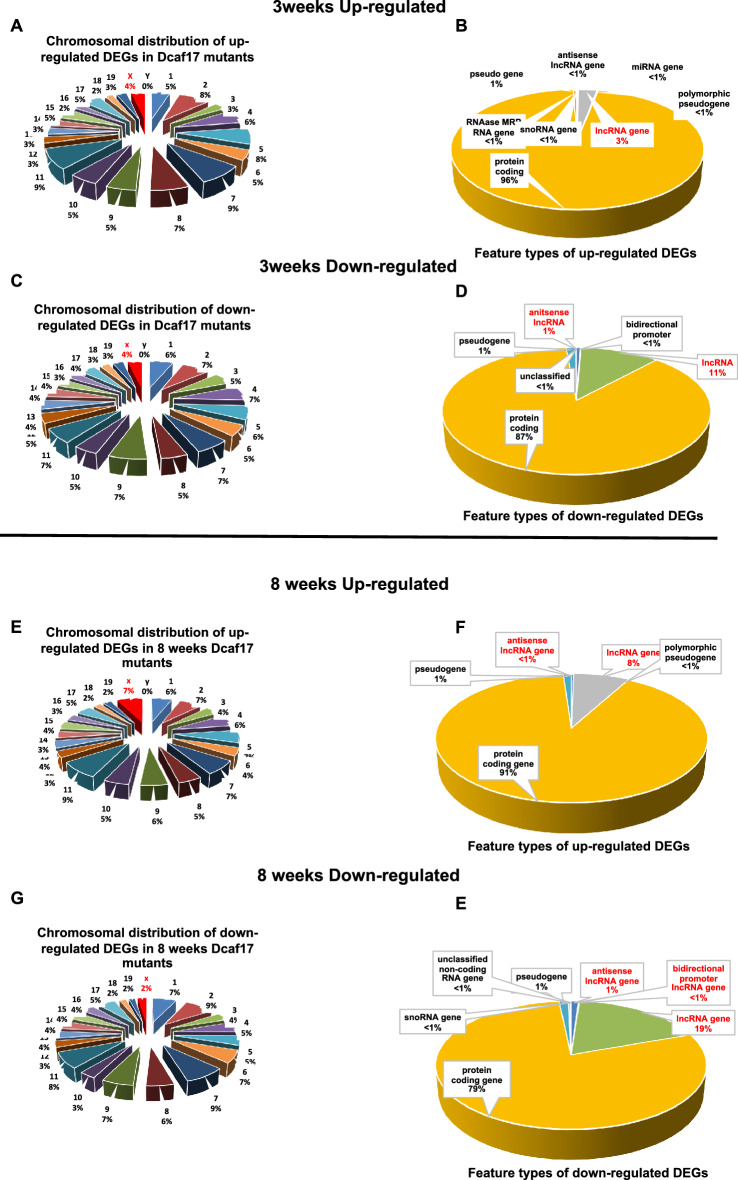
Chromosomal distribution (left) and feature types (right) of DEGs in testes of 3 weeks and 8 weeks old mice.(**A,C**) the DEGs are distributed on all the chromosomes but the Y-chromosomes. Note, in the 3 weeks age group lncRNAs are among the feature types of the DEGs and are higher in proportion among the down-regulated DEGs, (**B,D**). (**E,G**) in the 8 weeks age group the DEGs are distributed on all the chromosomes but the Y-chromosomes. Note, lncRNAs are among the feature types of the DEGs and are higher in proportion among the down-regulated DEGs, (**F,E**)**.**

The majority of our DEGs are protein coding genes, with some long non-coding RNAs (lncRNA), distributed among autosomal and sex chromosomes. 4% and 7% of up-regulated DEG are X-chromosome linked genes at 3- and 8-weeks, respectively. Moreover, 4% and 2% of down-regulated DEGs are X-chromosome linked genes at 3- and 8-weeks, respectively, (Fig. 2). This indicates impaired genetic regulation that might include impaired X-chromsome silencing. Long non-coding RNAs (lncRNA) were identified among the up- and down-regulated genes in the *Dcaf17* mutants (Fig. 2, Supplementary tables 2–4). In the 3-weeks age group, 38 (3%) lncRNAs were up-regulated, while 188 (11%) were down-regulated. In the 8 weeks age group, 156 lncRNA (19%) were down-regulated, while 141 lncRNAs (8%) were up-regulated. Collectively, aberrant lncRNA expression was higher among down-regulated genes compared to the up-regulated genes in both age groups.

### Enrichment of tissue specific genes among the DEGs in *Dcaf17* mutants

**Figure 3:**
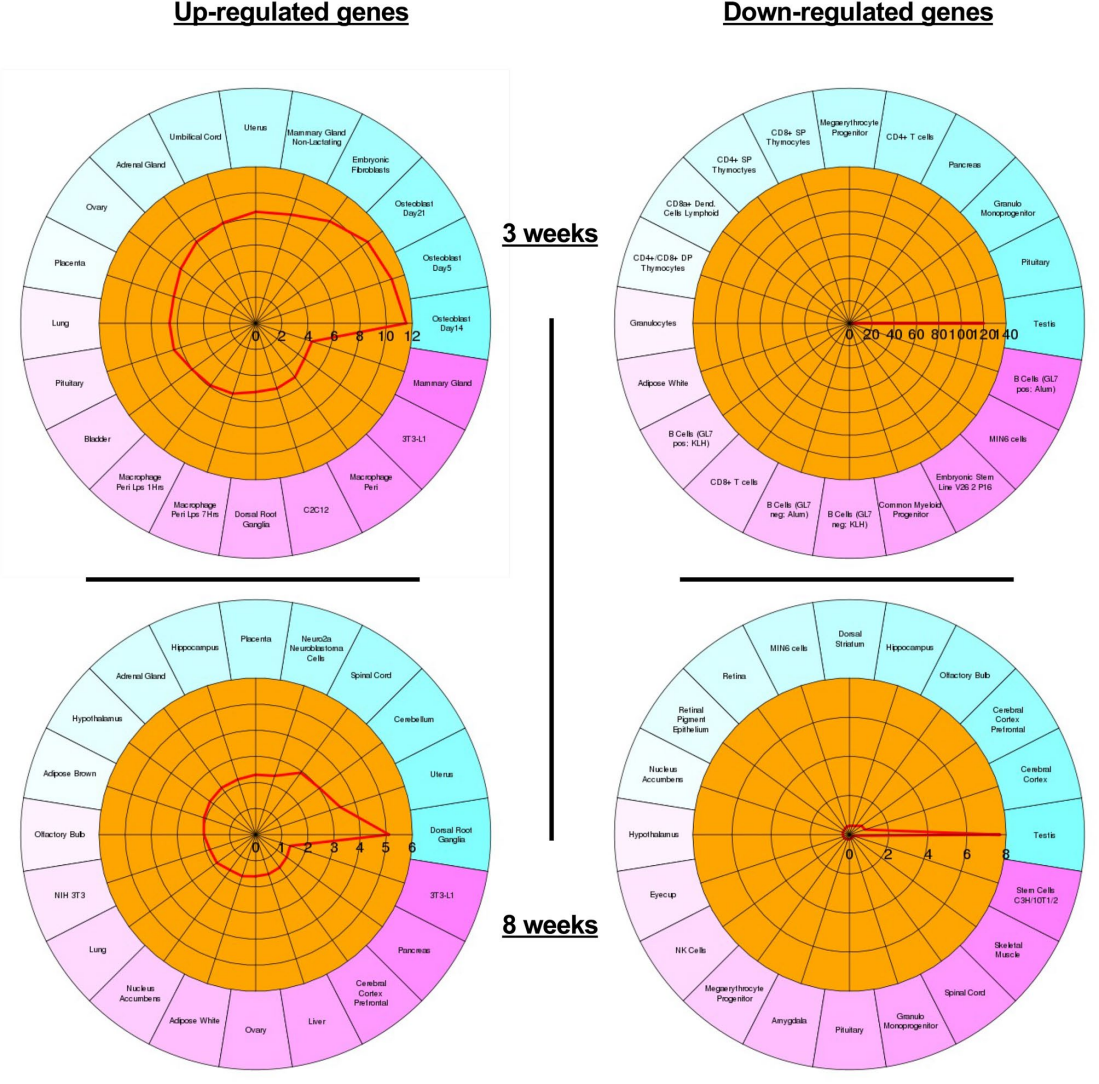
Cell type enrichment (CTen) analysis of the up-regulated and down-regulated genes. A minimum score of 2 is required to obtain confident cell type enrichment. In both age groups, the down-regulated genes point to testicular cells and the up-regulated genes point to a variety of somatic cell types like osteoblast, mammary gland, uterus, placenta, lung, bladder, dorsal root ganglia and others. B-cells GL7 neg./pos. Alum/KLH (with and without adjuvants Alum and KLH), Macrophages peri LPS 1 h and 7 h (1 and 7 hrsLPS challenged perinoteal macrophages).

To examine the DEG tissue expression pattern, the CTen, a cell type enrichment analysis, was used^13^. This web-based platform allows the identification of enriched cell types when supplied with high-throughput data. A minimum score of 2 is required to minimize the false positive rate. For both 3 and 8 weeks age groups, the down-regulated genes displayed a testis cell type enrichment, whereby the up-regulated genes enriched for several different cell types but testis (Fig. 3).

**Table 1:** DEGs associated with male reproductive phenotypes (MRP).

	**Male reproductive phenotype**	**Meiosis phenotype**
**3 weeks DEGs**		
Up-regulated	10%	7%
Down-regulated	12%	12.7%
**8 weeks DEGs**		
Up-regulated	7%	2.2%
Down-regulated	9%	13.4%

### Down-regulated genes are enriched for spermatogenesis associated processes

Spermatogenesis related genes, *Akap4*, *Cabyr*, *Theg*, and *Spata19*, which were identified amongst the top 11 DEG, were only found in the down-regulated group in the 3 weeks age group, but not in the 8 weeks age group (Supplementary Tables 2 and 3). These results suggest that *Dcaf17* is directly or indirectly involved in maintaining the expression of spermatogenesis-associated genes. Indeed, searching the Mouse Genome Informatics (MGI) database for known DEG phenotypes revealed that fewer genes were identified with a male reproductive phenotype (MRP) in the up- (10% (3 weeks); 7% (8 weeks)) than in the down-regulated (12% (3 weeks); 9% (8 weeks)) DEGs (Table 1, Supplementary Table 4). Notably, the proportions of meiotic associated genes were different amongst the MRPs and showed higher percentage (up to 13%) in the down-regulated DEG in both groups.

### Gene ontology analysis revealed that up-regulated genes are mainly implicated in gene transcriptional activities

**Figure 4:**
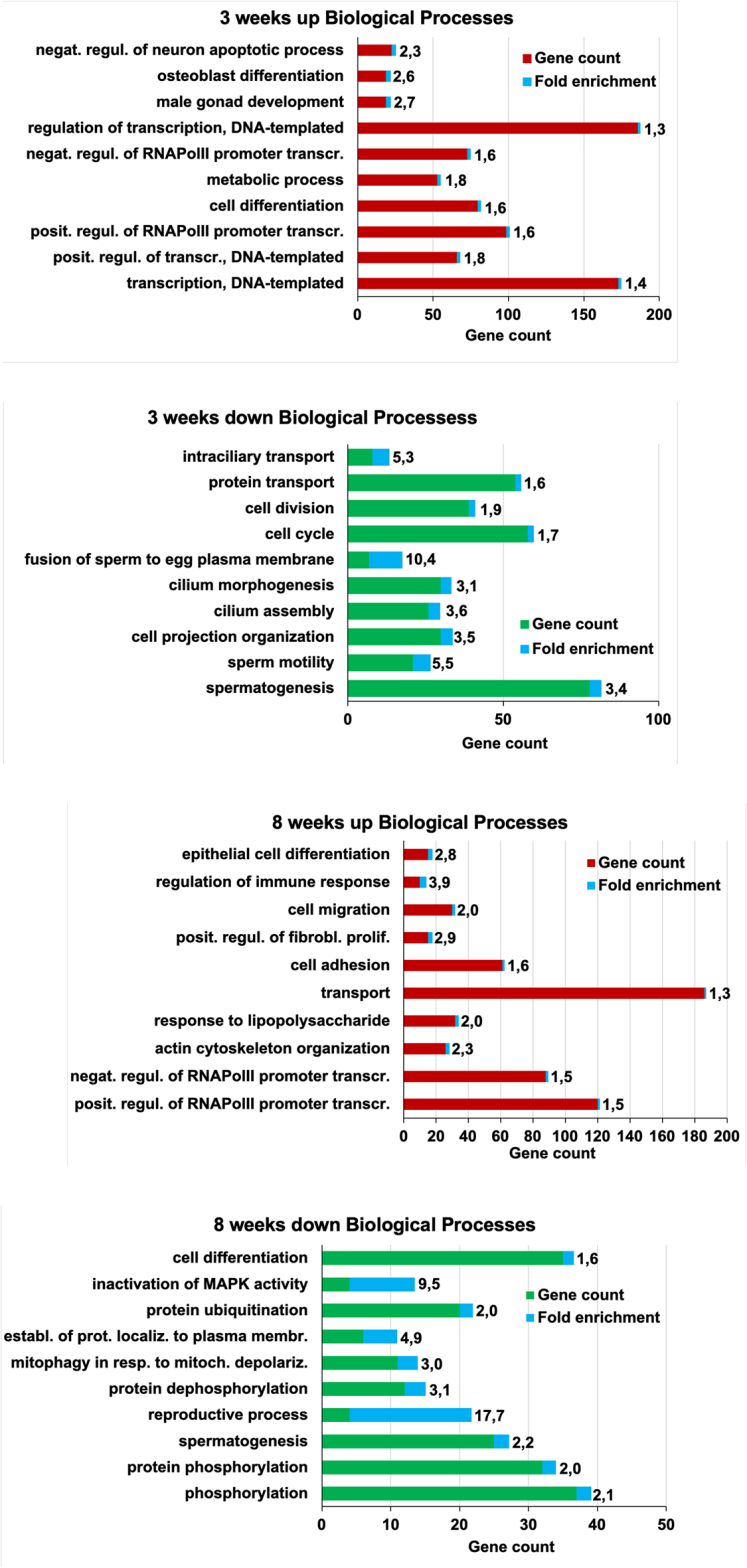
TOP 10 and TOP 11 Gene Ontology enrichment of biological processes of the up- and down-regulated DEGs of 3 and 8 weeks old murine testes. In red and green, up- and down-regulated genes, respectively. In blue and in numbers the fold enrichment. Note, the enrichment of spermatogenesis and male reproductive system associated processes in the down-regulated DEGs, while the up-regulated DEGs are enriched for a variety of somatic tissues and processes.

To have a clearer overview of what biological processes, molecular functions and cellular components might be altered due to the *Dcaf17* disruption, we followed up with a gene ontology (GO) enrichment analysis (Fig. 4, Supplementary Tables 2, 3 and 5) using the DAVID database^14^. We found that biological processes involved in transcriptional regulation are highly enriched in the upregulated genes at both, 3 and 8 weeks *Dcaf17* KO mice testes. Further, age specific biological processes are enriched in upregulated DEGs at 3 weeks such as cell differentiation and metabolic process and 8 weeks such as actin cytoskeleton organization and response to lipopolysaccharide. Molecular function terms are highly enriched for protein, actin and metal binding in both age groups. Several cellular components are highly enriched in upregulated DEGs in both 3 and 8 weeks group including extracellular exosomes, cytoplasm, nucleus, membrane, etc.

Collectively, molecular function enrichments strongly suggest dysregulation of transcriptional processes, which is in agreement of the enrichment for biological processes for the upregulated genes in response to *Dcaf17* disruption. In concordance with the reported male infertility phenotype in *Dcaf17* KO mice, the majority of biological processes in downregulated DEGs at 3 weeks are involved in male reproductive processes such as spermatogenesis, cell projection organization, sperm motility, and cilium morphogenesis. Also, reproductive processes, spermatogenesis, phosphorylation/ dephosphorylation, mitophagy, ubiquitination and other biological processes are highly enriched in downregulated DEGs at 8 weeks.

Molecular functions of downregulated targets in 8 weeks are mainly involved in enzymatic functions such as kinases, phosphatases, transferases and hydrolases. Nucleotide bindings including ATP and cAMP binding are enriched in this group as well. In the 3 weeks downregulated DEGs, molecular functions are enriched for ligases, transferases including ubiquitin-protein transferases and protein-cysteine S-palmitoyltransferase activity, urea channel activity, and ion, nucleotide and protein binding. Several cellular components are enriched in our dataset for both ages including cytoskeleton, cytoplasm, cell projection, cilia, and others. However, age specific cellular component such as acrosomal vesicle and sperm fibrous sheath are enriched at 3 weeks and the manchette was enriched at 8 weeks (Supplementary Tables 2, 3 and 5). In agreement with the CTen analysis, the up-regulated GO_TERMs showed that the up-regulated genes cover a wider range of processes than those of the down-regulated genes, which may suggests that Dcaf17 suppresses genes of somatic tissues in testis, while supporting the expression of spermatogenesis related genes.

### Effect of *Dcaf17* disruption on chromatin modulators and condensators

**Figure 5:**
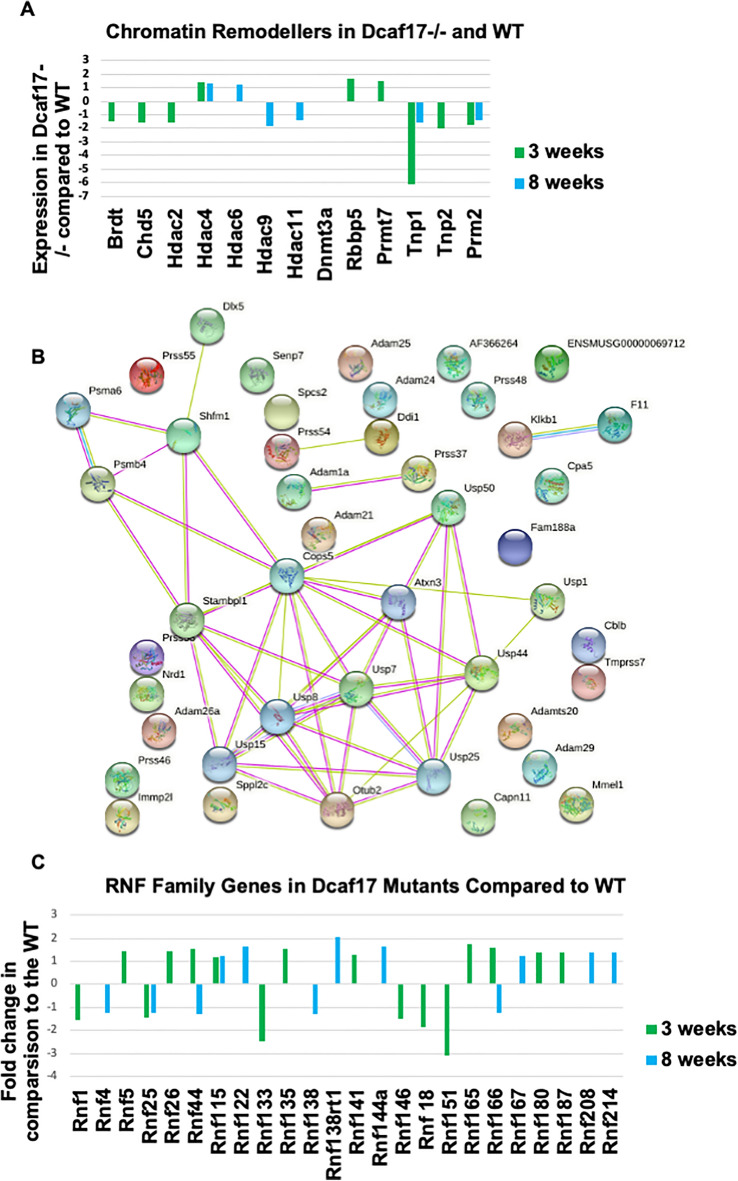
DEGs implicated in chromatin remodeling and proteolysis. (**A**) Gene expression alterations of chromatin re-modelers is altered in *Dcaf17* mutants. Changes are observed at enzymatic levels of epigenetic factors, but also at structural levels of the chromatin. The bars below zero are down-regulated and those above are up-regulated. Values of zero appear as missing bars. (**B**) String protein network analysis of 43 down-regulated and proteolysis associated genes the in Dcaf17 mutants. Note, experimentally verified central network is mainly involved in de-ubiquitination. 10 of these 43 factors are already known to display male fertility phenotypes. These factors are Prss37 and Prss55, Adam1A, Adam24, Adam26a, Mmel1, Sppl2c, Psma6, Cops5, Usp1. (**C**) Rnf E3 ligases that are dis-regulated in *Dcaf17* mutants. The Ring Finger Protein (Rnf) family, which is overwhelmingly composed of E3 ligases and found that a significant number is dysregulated in the *Dcaf17* mice. Some of which, however, occupy different functions like Rnf51, which is a major component of the PRC1 and is a histone modifier.

Our phenotypic analysis of *Dcaf17* KO mice sperm showed strong indications for aberrant chromatin condensation similar to the report of Takeda and colleagues^15^, which might have amongst other effects resulted in the abnormal sperm head morphology^11^. Therefore, we examined the expression profiles of chromatin modulators and condensators. Indeed, we found several genes that are differentially expressed in the mutant (Fig. 5A). Our results showed that some chromatin re-modellers are indeed dysregulated such as *Brdt*, *Chd5*, *Tnp1* and *Tnp2* and *Prm2*.

### Proteolytic and non-proteolytic ubiquitination pathways associated genes in *Dcaf17* KO mice

*Dcaf17* acts as a substrate receptor in the E3 ligase complex, and is putatively involved in ubiquitination proteolytic and non-proteolytic pathways. As shown by the DAVID and CTen analysis, the *Dcaf17* deletion caused up-regulation of genes which are normally expressed in somatic tissues, and contribute mostly to their development or their homeostasis. This perhaps means that protein degradation processes are heavily impaired, affecting their wide-ranging down-stream targets including proteins that are involved in transcription and/or mRNA stability as we have observed in our data (Fig. 5B, Supplementary Tables 2 and 3). This hypothesis is strengthened by observations that in 3 weeks old mutant testes, 43 proteolysis-associated genes were down-regulated, some of which exhibited a male reproductive phenotype such as Sppl2C, Immp2L, Usp1, Mmel1, Prss55, Prss54, Adam1A, Adam24 and Adam26A (Fig. 5B). These proteolysis-associated genes are mainly involved in de-ubiquitination. Their down-regulation may indicate that the proteolytic pathways and proteasome mediated protein degradation are compromised, which is supported by the additional UPS related genes that were detected (Supplementary tables 2 and 3). The UPS and proteolysis associated genes that were detected in the 8 weeks age group display mainly phenotypes in somatic tissues, while only a few are known to contribute to testicular tissues including the germ line. In addition, we analysed the Ring Finger Protein (Rnf) family, which is overwhelmingly composed of E3 ligases and found that a significant number is dysregulated in the *Dcaf17* mutant mice (Fig. 5C). Moreover, we found SMCL2, which itself is a germline-specific Polycomb Repressor Complex1 (PRC1) subunit, and an RNF2 interactor, to be 1.36 fold down-regulated in the 8 weeks age group. SCML2 prevents the murine germline from expressing somatic genes at later stages of spermatogenesis by mediating the ubquitination of H2A119K at transcription start sites. Paradoxically, it also prevents the H2A119K ubiquitination of meiotic sex chromosomes^16^.

phenotypes in somatic tissues, while only a few are known to contribute to testicular tissues including the germ line (Supplementary tables 2 and 3).

#### Male reproductive phenotype associated DEGs mapping to germline cell types

Our dataset analysis showed that many dysregulated genes, approximately up to 12% of DEGs, are known to have male reproductive phenotype (MRP). In order to get an insight into the potential roles of Dcaf17 on germ cell development and gene expression, we mapped these MRP genes using available mouse testis transcriptomics data of Green et al. and da Cruz et al.^17,18^. Using this approach, we were able to retrieve up to 77% and 93% of DEG-MRP at 3 weeks and 80% and 98% at 8 weeks in da Cruze and Green datasets, respectively. In both age groups and genotypes, DEGs that are associated with MRPs are expressed in wildtype mouse premeiotic (spermatogonia), meiotic (spermatocytes) and post-meiotic (spermatids) germ cells (Supplementary Figs. 1–8).

Furthermore, apart from some overlap, the majority of the clusters expressed in one cell type is absent in another. In mapping our targets to the dataset of Cruz and colleagues^18^, the down-regulated genes of both age groups, 3 weeks and 8 weeks, are also normally expressed in these cell types. However, the biggest portion is found to be expressed in round spermatids, while a large number of up-regulated genes were mainly expressed in premeiotic cells. In agreement with the data set of da Cruz, mapping targets to the Green data set, the majority of the down-regulated genes of the 3 weeks age group are normally expressed in the spermatids, while a smaller cluster is expressed in the spermatogonia and spermatocytes. A similar situation is found is for the down-regulated 8 weeks age group, with the exception that fewer genes are expressed in the spermatids in the WT in comparison to the 3 weeks group. However, smaller clusters, but with strong expression are found in the preleptotene spermatocytes and spermatogina among the downregulated DEGs at 8 weeks. The up-regulated DEGs in the both age groups are mapped to WT preleptotene and dispersed clusters among the other stages of spermatocytes and spermatids as well as with some strong expression in spermatogonia.

#### Expression of sertoli cell markers

Sertoli cells are the only somatic cells in the seminiferous tubules, and form the blood testis barrier via cell–cell interactions. They also provide structural and nurturing support for the spermatogenic cells, in addition to instructive cues that are being secreted to help orchestrate the complex process of spermatogenesis, reviewed in^19,20^. Given these essential functions in spermatogenesis, and in spite of our failure to detect *Dcaf17* expression in Sertoli wild type cells, we examined our data sets whether the Sertoli cells are affected by the Dcaf17 mutation, to probe for indirect and cell–cell communication effects (physical or paracrine). We compared our data sets with the data set of Zimmerman and colleagues^21^, who isolated the Sertoli cells with a Sox9 driven eGFP reporter from different ages (P5, P10, P18, P25, P35) and determined the expression profiles (Supplementary Fig. 9). In general, the heatmaps show that the *Dcaf17* mutants exhibit some similar expression tendencies to the early stages of P5 and P10, but not to the other stages. Specifically, we detected some Sertoli cell markers of different Sertoli specific processes, but never a significant number to claim a comprehensive profile. For example, we found the proliferative markers for immuature Sertoli cells at P5 *Cdk14*, *Mcm2, Mcm3, Mcm6* (3 weeks of the *Dcaf17* mutants) and Ccnd2, Mcm7 (8 weeks *Dcaf17* mutants), but not other markers to complete the profile like *Aurkb, Cables1, Ccnd3, Camk1, Cdc25a, Cdk2 Dlgap5, Klf4, Fam64a, Tpx2, Ckap2, Anln, Mcm5, Melk, Mki67* or *Skp2*. Another example, we did detect mature and proliferation inhibited Sertoli cell markers corresponding to the P10 age of the Zimmerman et al. data set, like the negative cell cycle regulators Dab2ip (3 weeks), but we did not find *Cdkn1a* and *b*, *Cdkn2a, Hmga1* or *Nkx3.1*. Similar were the cases for markers of the blood testis barrier, immature and mature markers, those which regulate the proliferation of spermatogonia, regulatory factors with paracrine effects. And although we found some upstream components of the Hedgehog pathway in the 3 weeks age group (*Dhh, Ptch1, Smo*), we did not detect down-stream components like (*Kif7, Sufu, Gli1,2 or 3, Zeb1, Zeb2*, etc.). Regarding the retinoic acid synthesis markers as well well as glucose and lactate metabolism, we did not find any marker genes being expressed.

## Discussion

Ubiquitin E3 ligases regulate many different cellular functions through ubiquitination of protein targets. Dcaf17 is an E3 ligase substrate specific receptor, deletion of which causes male mouse infertility due to low sperm count, abnormal sperm morphology and abolished motility^11^. To understand the underlying mechanism of this specific spermatogenic failure, we performed RNAseq (Ion AmpliSeq) based gene expression profiling, examining *Dcaf17* wild type and mutant testicular transcriptional profiles of 3 weeks and 8 weeks old mice. These were studied and analyzed separately without cross-comparison, i.e., 3 weeks WT vs 3 weeks KO; and 8 weeks WT vs 8 weeks KO. However, occasionally we did highlight pathways or biological processes within similar age groups between WT and KO, but without establishing causal relationships. In our study, we ascertained that several pathways were affected, including ubiquitination, proteolysis, cross-talk between E3 ligase families, regulation of transcription, chromatin remodelling and lncRNA. Collectively, these data strongly suggest that Dcaf17 impacts the proteome, which ultimately dysregulate testis transcriptome. Herein, RNA-Seq analysis showed that the reported infertility phenotype of Dcaf17 KO could be caused via disturbance of several mechanisms including protein proteolysis, chromatin modulation and gene expression.

### Proteolysis

Defects or down-regulation in the ubiquitin–proteasome proteolysis are associated with a variety of diseases like neurodegeneration, reviewed in^22,23^, cancer, reviewed in^24–26^, muscle atrophy, reviewed in^27^. Our data revealed 43 proteolysis-associated genes were down-regulated in the 3 weeks age group indicating that the *Dcaf17* mutation caused protein degradation impairment. This might directly cause the fertility defect, since 11 (~ 26%) out of these aforementioned 43 factors were reported to display male reproduction phenotypes (Table 2). Perhaps, disruption of Dcaf17 impairs proteasomal function, or potentially lack of Dcaf17 or Dcaf17-associated factors forces other E3 ligase complex members to resort to alternative transcriptional regulation as transcription factors or co-factors^28–37^.

**Table 2:** Shows 11 out of 43 proteolysis associated genes that are implicated in male reproduction.

Gene	Function/Phenotype	Reference
Prss37	Serine protease, defective sperm migration and sperm-egg interaction	^23^
Prss55	Serine protease, defective sperm migration in utero	^24^
Adam1a	Zinc metalloprotease, defective sperm migration in utero	^25^
Adam24	Metalloproteinase on sperm surface, polyspermic embryos at the pronuclear stage resulting in reduced fertility	^26^
Adam26a	Thought to play a role in spermatogenesis	^27^
Cops5	protease subunit of COP9 signalosome complex, acts as the catalytic center of the de-neddylation activity of cullins. Mutation leas to embryo growth arrest	^28^
Mmel1	Metalloendopeptidase, deficiency leads to impaired fertilization and aberrant embryo development	^29^
Immp2l	Inner mitochondrial membrane peptidase 2-like is required for signal peptide sequence processing of proteins that require mitochondrial import. The mutated gene disturbs the inner mitochondrial proteostasis and results in female and male infertility	^30^
Sppl2c	Signal peptide peptidase. Disruption leads to a partial loss of elongated spermatids, reduced motility and reduced litter sizes when mated with *Sppl2c* deficient female	^31^
Psma6	Component of the 20S core proteasome complex, has also been associated with low fertility	^32^
Usp1	De-ubiquitinating enzyme causes amongst others Fanconi anemia (chromosome instability), which implicates infertility of male mice	^33^

Thus, these are strong indications that this group of proteins is essential for spermatogenesis, which is associated with the cellular proteolytic degradation system and that the expression of this group is regulated directly or indirectly by Dcaf17. While more experimental examinations will shed more light on this matter, the genes known to be associated with spermatogenesis (11 out of 43) in Table 2 are not involved in early-, but in late-spermatogenesis, sperm physiology up to fertilization, early embryogenesis and erectile dysfunction. However, together with our previous report, in which we describe that the *Dcaf17* disruption caused a major histological alteration at 23 dpp^11^, we believe that *Dcaf17* plays a greater role in early spermatogenesis.

### Cross-talk with other E3 ligases

Our data suggest critical roles of *Dcaf17* in general intracellular protein degradation using the proteolytic ubiquitination pathway, which is essential for normal male germ cell development. *Fbw11 (ß-TrCP)*, a member of an E3 ligase complex, is required for a successful mitosis-meiosis transition of spermatogonia, *requiring* down-regulation of *Dmrt1* for mitotic proliferation of spermatogonia^38^. We found *Fbw11* was 1.56-fold up-regulated in 3 weeks old *Dcaf17* mutants and *Dmrt2*, a family member of *Dmrt1*, being 1.64-fold up-regulated in 8 weeks old mutants. Furthermore, Rnf family members, partly down-regulated in our dataset, are overwhelmingly composed of E3 ligases and believed to control the stability, trafficking and activity of proteins^39,40^. The Rnf family are examples of E3 ligases that function proteolytically and also in non-proteolytic pathways.

### Regulation of transcription

The most abundant genes that were up-regulated in both mutant age groups, at 3 and 8 weeks, were transcription regulators (mostly RNA pol II). Dcaf17 appears to suppress expression of these. In the 3 weeks age group, 78 genes were down-regulated, and 41 up-regulated. Interestingly, the 41 up-regulated genes were mainly gene expression regulators, signalling molecules, chromatin modulators, and apoptosis regulators. Presumably these are negative regulators in this developmental context. The 78 down-regulated genes, however, were structural, physiological and “organisational” spermato- and spermiogenesis genes, involved in structural aspects of spermiogenesis^41–43^ potentially explaining the aberrant sperm head and flagellar structures observed in *Dcaf17* KO phenotypes^11^.

Notably, the 3 weeks age group highlighted more male fertility relevant biological processes than the 8 weeks age group. A plausible explanation could be that *Dcaf17* ‘s primary importance is developmental (i.e., first wave of spermatogenesis), namely to establish the functional germ line. This is in line with our observations that the first histological changes appear at 10–14 dpp in the *Dcaf17* mutant^11^. Alternatively, the severity of testicular phenotype is higher at 8 weeks than at 3 weeks of age, manifested by loss of germ cells, which makes capturing reproductive related genes more challenging in our analysis.

### Dcaf17 might play a role in pre-meiosis

The gene enrichment analysis identified many important spermatogenesis events that are acquired across germ cell types. Moreover, fertilisation and egg activation associated genes were also highlighted in our dataset, supported by mapping the identified DEGs that are associated with male reproductive phenotypes to the data sets of da Cruz et al. and Green et al., which strongly indicate that Dcaf17 plays a role in regulating gene expression in spermatogonia, spermatocytes and spermatids^17,18^. It is interesting to note that many genes, which are involved in meiosis were dysregulated at both ages, suggesting that Dcaf17 may play a role in establishment of the germ line in testicular development as well as affecting subsequently post-meiotic sperm development. Although we have shown that *Dcaf17* mutant mice have normal chromosomal synapsis during pachytene stage in comparison to wildtype^11^, it is not clear, whether Dcaf17 has any role in meiosis entry and/or exit processes. Together, with the fact that round spermatids and further developed sperm are transcriptionally inactive, it is tempting to speculate that Dcaf17 is also involved in the regulation of synthesis, storage or maturation of mRNA and/or protein that is required at later in the sperms development or in the female reproductive tract.

### Chromatin remodelling

The *Dcaf17* mutation has caused amongst other sperm phenotypes an abnormal head sperm morphology suggesting abnormal chromatin architecture^11^. Indeed, our RNA-seq study suggests that chromatin modulators and chromosome condensation associated genes are significantly altered. *Brdt*, loss of which resulted in failure of germ cell development^44–46^, was 1.5-fold down-regulated in 3 weeks old *Dcaf17* deficient mice. *Hdac2*, a Chd5’s interaction partner, was also down-regulated. Strikingly, this is supported by the simultaneous down-regulation of several genes that are responsible for chromosome condensation and replacement of histones by protamines, all of which are physical interaction partners according to the STRING database. Deletion of *Tnp1*, *Tnp2, Prm2, Odf1, Smcp* and *H1fnt* genes resulted in similar phenotypes like *Dcaf17* KO including reduced or abolished fertility, dysmorphic sperm, reduced sperm number and motility and impaired chromatin condensation^47–54^. Indeed, several reports have highlighted the critical role of replacing histones with protamines to ensure efficient chromatin condensation and packaging as essential requirement yielding normal sperm function^47–49,53,54^.

Additional epigenetic factors such as Hdacs and DNA methyltransferase *Dnmt3a* were also altered in 3 weeks old mice, including histone methyltransferase *Rbbp5* and arginine/histone methlytransferase *Prmt7*, suggesting that chromatin modifications in *Dcaf17* mutants might be severely altered and could potentially be a major cause for the *Dcaf17*^*−/−*^ infertility through impaired chromosome condensation. SMCL2, a Polycomb Repressor Complex1 subunit, was down-regulated in the 8 weeks age group. *SCML2* knock-out mice display a similar phenotype as the *Dcaf17* knock-out, which is the up-regulation of somatic genes^11,16^, potentially indicating a positive feed-back mechanism.

### lncRNA

Long non-coding RNAs (lncRNA) were identified among the up- and down-regulated genes in the *Dcaf17* mutants in both age groups 3 weeks and 8 weeks. lncRNAs have several functions such as gene silencing^55^, X-inactivation^56^, imprinting and development^57,58^, which they can execute based on their ability to interact with proteins, but also with nucleic acids, reviewed by Wang and Chang^59^. Moreover, testicular lncRNA have been reported to play important roles in spermatogenesis of various eukaryotes including Drosophila, sheep and mice^60–62^. The levels of lncRNA, *Pldi* (polymorphic derived intron containing), were reduced in *Dcaf17* KO mice at both, 3- and 8-weeks of age. Indeed, Heinen and associates reported that *Pldi* KO mice display a reproductive phenotype manifested by reduced testis weight and sperm motility^63^. Certainly, further studies are required to identify the specific roles of the lncRNAs within the context of the Dcaf17 model and spermatogenesis in general.

### Sertoli cells

We failed to detect *Dcaf17* expression using immunohistochemistry in Sertoli cells. Nevertheless, the expression of Dcaf17 in Leydig cells and in the spermatogenic cells could affect the Sertoli cell on a functional level. The comparison of the 3 weeks and 8 weeks *Dcaf17* KO data sets with the P5, P10, P18, P25 and P35 revealed some similar tendencies to the ages P5 and P10 based on the heatmaps, but when examining for individual profiles like proliferation of immature Sertoli cells or blood-testis barrier, we failed to reconstruct these profiles in our *Dcaf17* knock-out model. A plausible explanation might be that the underrepresentation of the Sertoli cells is masked by the vast number of spermatogenic cells. The other explanation could simply be that the Dcaf17 knock-out has no effect on the Sertoli cells. Anyhow, the comparison regarding the ages, although close, does not match. Therefore, further experiments are required, which compare isolated and enriched Sertoli cells from the wild type and the Dcaf17 mutant from the same age groups to shed more light on this issue.

Finally, our study describes the effects of the Dcaf17 mutation, on transcriptional level, however, to address the functional ramifications of Dcaf17 disruption, we are performing germ cell specific deletion studies.

## Conclusion

*Dcaf17* is critical for fertility and its mutation causes the Woodhouse-Sakati Syndrome in humans. In our *Dcaf17* mouse model, male infertility is defined by an abnormal morphology, reduced motility, and low sperm count. Taken together, *Dcaf17*, an E3 ligase substrate receptor, appears to be involved in the regulation of many different processes that are critical for normal spermatogenesis in mice.

## Material and methods

### Animals

Mice carrying the *Dcaf17* mutation (KO) and C57BL/6 J (WT) were obtained as previously described^11^. All animal handling and usage were subjected to ethical approval from Animal Care and Use Committee at King Faisal Specialist Hospital and Research Centre, RAC#2,160,019. Animals were housed under 12 dark/light cycle with free access to rodent chow and water till time of tissue harvesting.

### Total RNA isolation and cDNA synthesis

Testicular tissues of 3 weeks and 8 weeks old *Dcaf17* WT and KO mice were collected and snap frozen in liquid nitrogen. Total RNA was isolated using the QIAGEN RNeasy mini kit and treated with DNase I according to the manufacturer’s protocol (Qiagen, Valencia, CA, USA). The RNA quality was verified on agarose gels. Reverse transcription was performed on total RNA from using Superscript III First Strand Synthesis system (Life Technologies, ThermoFisher Scientific, Waltham, MA, USA) as per manufacturer’s protocol. Briefly, 5 µg of total RNA, 50 µM oligo(dT), 10 mM dNTP mix and RNAse-free water were mixed in a 10 µl total reaction volume. The mixture was then incubated at 65 °C for 5 min followed by incubation on ice for 5 min. Meanwhile the cDNA synthesis mixture was prepared, which consisted of 2 µl of 10X RT buffer, 4 µl of 25 mM MgCl2, 2 µL of 0.1 M DTT, 1 µl of RNaseOUT (40 U/µl) and 1 µl of SuperScript III RT. Then the cDNA synthesis mix was added to the RNA-primer mix. The combined solution was gently mixed, collected by pulse centrifugation and incubated for 50 min at 50 °C. Subsequently, the reaction was terminated at 85 °C for 5 min and then placed on ice. Condensation was again collected by pulse centrifugation. 1 μL of RNAse H was added and incubated for 20 min at 37 °C. Finally, the cDNA synthesis reaction was then stored at − 20 °C.

### Quantitative-PCR (Q-PCR)

To set up a Q-PCR reaction, 2 µl of 1:10 diluted tissue cDNA was used as template in a 20 µl of total reaction mixture for each cDNA sample. The reaction mixture contained 12.5 µl, SYBER Green Supermix (BIO-RAD Laboratories, Hercules, CA, USA) and 1 µl of 100 nM of each forward and reverse primer. For each sample, a parallel reaction was setup using GAPDH primers as endogenous control. The Q-PCR was carried out using the Applied Biosystems AB7500 thermal cycler, ThermoFisher Scientific, Waltham, MA, USA). The reaction steps were heat activation at 95 °C for 15 min followed by 40 cycles of denaturation at 95 °C for 30 s, primer annealing at 60 °C (unless otherwise specified) for 30 s, and primer extension at 72 °C for 30 s, followed by melting curve analysis (55 to 95 °C; in 0.5 °C increments) to verify specificity of PCR products. All PCR products were also run in ethidium bromide stained agarose gels to verify the presence of single bands. All genes were analysed as biological triplicates (n = 3) and in technical triplicates from each biological sample. GAPDH was used as an internal housekeeping control, and data were displayed as LOG2 fold change of the 2^−ΔΔCt^ method. The primers used in this study are in Supplementary Table-Primers.

### Ion AmpliSeq transcriptome sequencing

The Ion AmpliSeq Transcriptome Mouse Gene Expression protocol (P/N A36553, ThermoFisher) was used to generate Ion AmpliSeq libraries from total RNA (10-15 ng per samples). Standard kit conditions for template preparation on the Ion Chef and sequencing on the Ion Proton instrument using the Ion P1 Sequencing 200 V3 kit (P/N 4,488,315, ThermoFisher) were followed. All libraries were generated and sequenced on the Ion Proton P1 sequencing chips at the Laboratory for Biotechnology and Bioanalysis at Washington State University, Pullman, WA.

### Sequence reads and differential gene expression analyses

The primary analysis for AmpliSeq sequencing data of 12 samples was performed using Torrent Suite software and the AmpliSeqRNA plugin. Ion AmpliSeq RNA normalization for of each sample is calculated by the plug-in as the number of reads mapped per gene per million reads mapped (RPM). Three biological replicates for each group of testicular tissues of 3 weeks and 8 weeks old Dcaf17 KO mice as well as wild type were performed. The normalized data is subsequently analysed using PARTEK Genomics Suite (Partek Inc., St. Louis, MO, USA). After analysing the QC statistics, correlation plots, and principal component analyses (PCA), three samples were of poor quality and excluded from the subsequent analysis. The differentially expressed genes (DEGs) were identified using Analysis of Variance (ANOVA) with a p-value of < 0.05. The common and specific genes between 3 week- and 8 week-old mice were identified using Venn diagram approach and depicted using the online tool Venny2.0 (https://bioinfogp.cnb.csic.es/tools/venny/index2.0.2.html)^64^. The hierarchical clustering using Pearson’s correlation with average linkage clustering was performed by Multi Experiment Viewer (MeV4.0)^65,66^.

### Gene ontology and pathway analyses

Functional and gene ontology analysis of DEGs were performed using the database for annotation, visualization and integrated discovery (DAVID)^14^ and KEGG pathways. 1390 up-regulated differentially expressed genes (DEGs) and 1741 down-regulated DEGs of the 3 weeks age group were uploaded in DAVID as well as 1921 up-regulated and 903 down-regulated DEGs of the 8 weeks age group. All genes were differentially expressed with a *p-*value of < 0.05. Results were retrieved and analysed.

### Reproductive phenotyping and testicular expression profiling

The Mouse Genome Informatics (MGI, http://www.informatics.jax.org/)^67^ database was screened for phenotypic data regarding DEGs with *p-*value < 0.05 in 3 and 8 weeks Dcaf17 WT and KO mouse datasets. Genes with known reproductive phenotypes including male reproductive phenotypes such as male infertility, spermatogenic and testis defects, male meiosis, etc. were compiled. Publically available RNASeq datasets from Green et al. (Accession: GSE112393), da Cruz et al. (Accession: PRJNA317251) and Zimmermann et al. (GSE59698)^17,18,21^ were used to examine testicular cell-specific expression of DEGs that exhibited male reproductive phenotypes as previously described^10^.

### Protein–protein interactions of differentially expressed genes

Protein–protein interactions were analysed using the STRING database applying experimental data, textmining and co-expression. The STRING database hosts known and predicted protein–protein interactions^68^. Currently, the STRING database covers 24,584,628 proteins from 5,090 organisms (https://string-db.org/). The protein association were known interactions based on experiments and from curated databases as well as co-expression, text mining, and predicted interactions.

### Cell type enrichment (CTen) tool

The CTen is an online platform (http://www.influenza-x.org/~jshoemaker/cten/) to identify enriched cell types of target genes. DEGs list of 3 and 8 weeks’ time points, we used where a minimum score of 2 is the cut off enrichment score^13^.

### Statistical analysis

The statistical analysis for the Q-PCR are represented as the mean ± standard error of the mean (SEM). All the experiments were performed in triplicates of three biological replicates each. All statistical tests were two-sided and *p-*value < 0.05 was considered statistically significant. Statistical analyses were performed with SAS version 9.4 (SAS Institute Inc, Cary, NC) and PARTEK Genomics Suite (Partek Inc., St. Louis, MO, USA).

### Ethics approval and consent to participate

All animal handling, usage and experiments were subjected to ethical approval from Animal Care and Use Committee of the King Faisal Specialist Hospital and Research Centre (KFSHRC), RAC#2160-019, and were carried out in accordance with the KFSHRC guidelines and regulations. All methods are reported in accordance with ARRIVE guidelines (https://arriveguidelines.org) for the reporting of animal experiments.

## Supplementary Information

Supplementary Figure 1.

Supplementary Figure 2.

Supplementary Figure 3.

Supplementary Figure 4.

Supplementary Figure 5.

Supplementary Figure 6.

Supplementary Figure 7.

Supplementary Figure 8.

Supplementary Figure 9.

Supplementary Table 1.

Supplementary Table 2.

Supplementary Table 3.

Supplementary Table 4.

Supplementary Table 5.

Supplementary Table 6.

Supplementary Table 7.

Supplementary Table 8.

## Data Availability

The datasets generated and analyzed during the current study are available under this link: https://www.dropbox.com/s/yp74j2j2rpuij0k/AmpliSeq_Aligned_normalized_data_allsamples_analyzed.xlsx?dl=0
