# Supplementary material for: Disruption of male fertility-critical Dcaf17 dysregulates mouse testis transcriptome
Source: Sci Rep. 2022 Dec 12;12:21456. doi: 10.1038/s41598-022-25826-7 (PMC9744869; doi:10.1038/s41598-022-25826-7)
Supplement: Supplementary file 6 — Supplementary Figure 6. [file 41598_2022_25826_MOESM6_ESM.pdf]

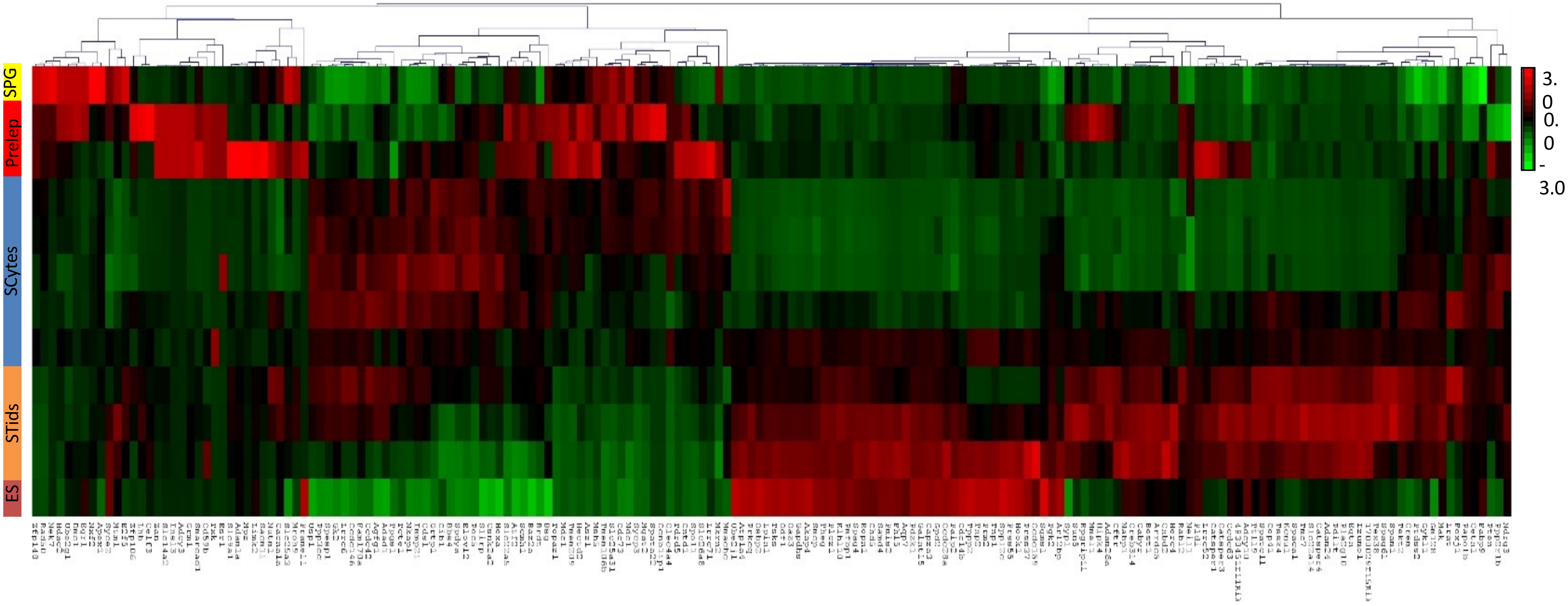

Supplementary Figure 6: Unsupervised hierarchical clustering of Green et al.'s dataset using 3 weeks DEGs (down-regulated) with male reproductive phenotypes. 182 genes found in Green *et al* dataset. Samples are denoted in rows and genes are denoted in columns. Red and green denote highly and weakly expressed genes, respectively.
