# Supplementary material for: Disruption of male fertility-critical Dcaf17 dysregulates mouse testis transcriptome
Source: Sci Rep. 2022 Dec 12;12:21456. doi: 10.1038/s41598-022-25826-7 (PMC9744869; doi:10.1038/s41598-022-25826-7)
Supplement: Supplementary file 9 — Supplementary Figure 9. [file 41598_2022_25826_MOESM9_ESM.pdf]

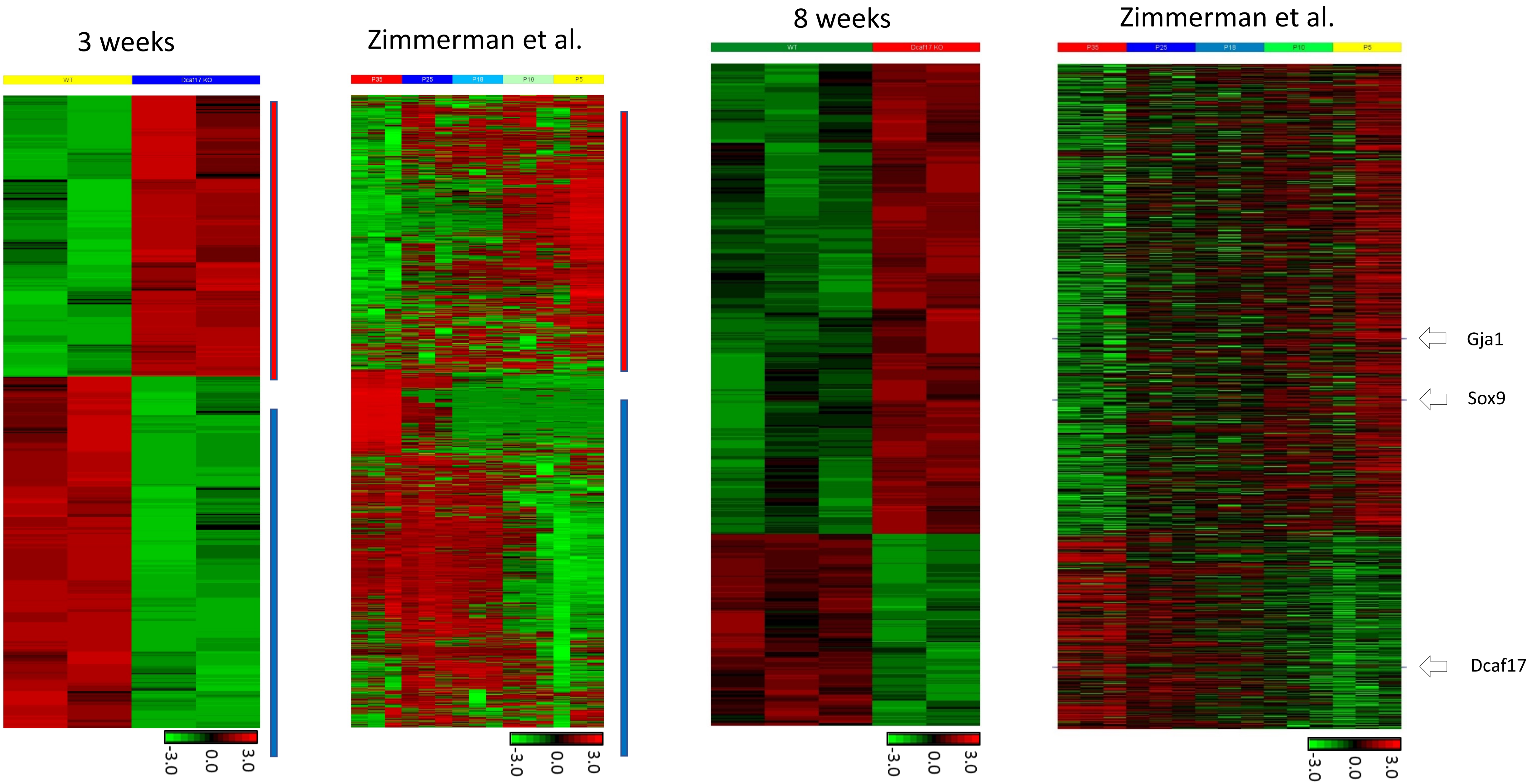

Supplementary Figure 9: Heatmaps comparing the 3 weeks and 8 weeks *Dcaf17* mutants with the data sets of P5, P10, P18, P25 and P35 from Zimmerman et al. Color code in the 3 weeks and 8 weeks old (yellow = Dcaf17 mutants), Zimmerman et al. (red = P35, dark blue = P25, light blue = P18, light green = P10, yellow = P5). For the heatmaps on the right, the Sertoli cell markers *Sox9* and *Gja1* (*Connexin 43* involved in gap junction formation) as well as *Dcaf17* are highlighted.
